# Risk factors for Carbapenem-resistant Enterobacterales infections: A case-control study

**DOI:** 10.4102/safp.v67i1.6029

**Published:** 2025-02-07

**Authors:** Sibongakonke Mbele, Sandeep D. Vasaikar

**Affiliations:** 1 Department of Laboratory Medicine and Pathology, Faculty of Medicine and Health Sciences, Walter Sisulu University, Mthatha, South Africa

**Keywords:** Carbapenem-resistant Enterobacteriaceae (CRE), Carbapenem-resistant Enterobacterales (CRE), CRE infections, CRE risk factors, carbapenemase genes

## Abstract

**Background:**

Over years, concerning Carbapenem-resistant Enterobacterales (CRE) have advanced globally, posing a major threat to global health. Prior studies highlight previous antibiotic use and prolonged hospital stays as paramount risk factors for CRE infections. However, there are limited reports available with a focus on identifying risk factors for CRE infections by comparing CRE cases with controls. The aim is to evaluate factors associated with CRE infections among individuals admitted to hospitals in Mthatha.

**Methods:**

A retrospective case-control study among patients who attended Nelson Mandela Academic Hospital (NMAH) and Mthatha Regional Hospital (MRH), Eastern Cape, South Africa. Demographic, medical history and current hospitalisation factors were captured on clinical research forms. GraphPad Prism version 8 software was used for statistical analysis.

**Results:**

Out of the 226 participants with CRE infection, CRE cases were more likely than controls to be adults (51.9%, odds ratio [OR]: 1.34, 95% confidence interval [CI]: 0.72–2.55) and of male sex (54.9%, OR: 1.48, 95% CI: 0,87–2,45). Significant risk factors for CRE infections included underlying illnesses (OR: 2.55, 95% CI: 1.41–4.60, *p* = 0.002), urine catheterisation (OR: 5.40, 95% CI: 1.45–18.33, *p* = 0.01), intravascular devices (OR: 2.48, 95% CI: 1.06–6.03, *p* = 0.05) and prolonged hospital stay (OR: 1.87, 95% CI: 1.01–3.39, *p* = 0.048). CRE cases compared to controls were almost twice as likely to demise or have an extended hospital stay of more than one month. *Klebsiella pneumoniae* (62.6%) and *Enterobacter cloacae* (60.6%) were prevalent Enterobacterales associated with CRE.

**Conclusion:**

Significant risk factors for CRE infections are underlying illnesses, urine catheterisation, intravascular devices and prolonged hospitalisation.

**Contribution:**

The complicated nature of CRE infections highlights the importance of targeted interventions to mitigate their spread and impact on public health.

## Introduction

Over time, there has been a significant increase in Carbapenem-resistant Enterobacterales (CRE) on a global scale.^1^ Carbapenem-resistant Enterobacterales and its related risk factors are of clinical significance in hospital environments, posing a substantial risk of developing CRE infections in hospitalised patients and contributing to increased rates of morbidity and mortality because they are easy to transmit from one individual to the other.^2,3^

Bacteria have evolved strategies to inactivate carbapenem-class antibiotics, and these organisms are known as CRE. Carbapenem resistance has emerged among Enterobacterales by virtue of two mechanisms. The first mechanism involves the acquisition of specific enzymes that hydrolyse carbapenems, called carbapenemases, such as *Klebsiella pneumoniae* carbapenemase (*bla*_KPC_) (class A), the metallo-β-lactamases (MBLs), such as the Verona integron-encoded metallo-β-lactamases (*bla*_VIM_) and New Delhi metallo-β-lactamases (*bla*_NDM_) (class B), oxacillinase-type carbapenemases such as the oxacillinase-48-type carbapenemases (*bla*_OXA-48_) and its variants (class D).^4^ The second mechanism involves the combination of extended-spectrum β-lactamase (ESBL) and/or AmpC production along with alterations in porin synthesis, either alone or in conjunction with overexpression of genes encoding efflux pumps.^5,6,7^

In South Africa, there have been limited active surveillance studies conducted on CRE.^8^ Similarly, in other parts of sub-Saharan Africa, there is a scarcity of active surveillance for CRE. African countries such as Morocco, Kenya and South Africa have reported *bla*_OXA-48_ as a predominant carbapenemase gene, followed by *bla*_NDM-1_.^8,9^

Patients exposed to CRE and subsequently colonised are at risk of developing CRE infections such as hospital-acquired pneumonia (HAP), bloodstream infections (BSIs), complicated urinary tract infections (cUTIs), ventilator-associated pneumonia (VAP), complicated intra-abdominal infections (cIAIs), wound infections and meningitis.^10^ The respiratory tract, urinary tract, bloodstream and abdominal cavity are among the most common sites for CRE infections in the body.

Previous studies have reported previous antibiotic exposure, admission in high-risk wards, prolonged hospital stays, age, use of invasive procedures (mechanical ventilation, surgery, tracheostomy, urinary or intravenous catheterisation, etc.), underlying diseases and previous hospitalisation history as significant risk factors for CRE infections.^2,3,11,12,13^

Few studies in South Africa have specifically focussed on comparing risk factors for the development of CRE infections through matching CRE cases and controls rather the focus was on CRE prevalence and CRE encoding genes. Carbapenem-resistant Enterobacterales pose a significant global health challenge because they are reported from various countries. Therefore, conducting comprehensive analyses of CRE infection, including prognosis, screening CRE risk factors and implementing effective prevention measures, would help mitigate incidences of CRE infection, reduce the risk of infection among inpatients and improve patients’ clinical outcomes.^14^

Reserve drugs are used for the treatment of CRE; these are colistin and tigecycline. Treatment of CRE depends on the infection site, isolated pathogen, resistance profiles and species-specific inherent resistances. Antibiotics that may have activity against CRE include carbapenems, polymyxins, aminoglycosides, tigecycline, fosfomycin and beta-lactam/beta-lactamase inhibitors (BLBLI).^15^

By employing a case-control study design and utilising advanced molecular methods for bacterial identification and characterisation, this research offers a comprehensive and detailed analysis of the risk factors underlying CRE infections in this region. This present study compared participants with CRE designated as cases against those with carbapenem-susceptible Enterobacterales (CSE) designated as controls to identify risk factors and assess their significance in the development of CRE infections within our population. This study aimed to identify the risk factors present in Nelson Mandela Academic Hospital (NMAH) and Mthatha Regional Hospital (MRH). The findings from this research have the potential to inform policymaking, guide clinical practice and enhance infection prevention and control measures in healthcare settings, thereby benefiting both individual patients and the broader community.

## Research methods and design

### Study design

The present study was a retrospective case-control study focussing on CRE bacterial isolates. The aim was to identify and compare risk factors associated with the development of CRE infections among participants who received medical care at NMAH and MRH between April 2019 and December 2020. This is a sub-study of the study: ‘Epidemiology, risk factors and molecular analysis of CRE in Mthatha, Eastern Cape, South Africa’. No direct intervention or interaction with any participant occurred as previously unidentified records of CRE data were used. Cases and controls were matched by hospital, ward and age for the determination and analysis of risk factors associated with the development of CRE infections. Participants with isolates that are carbapenem-non-susceptible and extended-spectrum cephalosporin-resistant (ceftriaxone, ceftazidime, ceftizoxime and cefotaxime) to *Escherichia coli (E. coli), Enterobacter aerogenes (E. aerogenes), Enterobacter cloacae (E. cloacae) complex, Klebsiella pneumoniae (K. pneumoniae), Klebsiella oxytoca (K. oxytoca), Raoultella species* fulfilling the CRE definition (CDC, 2015)^16^ were designated as CRE cases, while those with carbapenem-sensitive Enterobacterales (CSE) were designated as controls. Identification and analysis of CRE causative organism(s) together with potential risk factors were conducted. In this study, written informed consent was obtained from all adult cases and controls, while parental consent was obtained for child cases and controls. Participants were interviewed by a medical practitioner and a research assistant. A clinical research form (CRF) questionnaire was completed after obtaining the patient’s consent.

Factors associated with a clinical sample positive for CRE and CSE in participants who received medical care in NMAH and MRH were studied using a case-control study. Participants prospectively identified by the National Health Laboratory Service (NHLS) Microbiological laboratory (Mthatha branch) of each participating hospital with a clinical sample yielding CRE or CSE were eligible for the study and were selected from the laboratory register of the same hospital.

### Study population, setting, inclusion and exclusion criteria

The study on CRE cases and controls was conducted in the Eastern Cape province of South Africa, specifically in the Mthatha region, from April 2019 to December 2020. Data for the study were retrospectively obtained from the NHLS in Mthatha, focussing on bacterial isolates from patients receiving medical care at NMAH and MRH. These hospitals, located in the King Sabata Dalindyebo Local Municipality, house a wide range of departments, including the Emergency Department, Paediatric Ward, Maternity Ward, Obstetrics and Gynaecology, Outpatients Department, Surgical Services, Medical Services, Operating Theatre, Central Sterile Supply Department (CSSD) Services and the Ophthalmology Outpatients Clinic. The study population included all hospitalised patients who attended NMAH and MRH during the specified period, targeting CRE isolates while adhering to specific inclusion (all CRE isolates from patients encompassing non-duplicated bacterial isolates from different specimens and duplicated isolates from different specimens with different antibiograms) and exclusion (any organisms outside the Enterobacterales family, any Enterobacterales species other than CRE, and duplicated isolates from different specimens with the same antibiogram) criteria. In 2019, the Eastern Cape had an estimated human immunodeficiency virus (HIV) prevalence of 15.1% among adults aged 15–49 years, and this placed the province among those with high prevalence rates nationally.

### Sampling method and size

Participants with isolates that were Carbapenem-resistant and extended-spectrum cephalosporin-resistant were considered for the selection of cases. Participants with isolates that were carbapenem-sensitive from the same study base, same source population as cases and identified during the same period as cases were considered for the selection of controls. The study size was derived from the total number of participants available from a prospective cohort study implemented in hospitals and clinics in Mthatha and surrounding areas between April 2019 and December 2020. The sample population comprised participants with isolates (specimens – blood, urine, sputum, pus/rectal swab, arterial catheter tip, tracheal aspirate) that were Carbapenem-resistant and extended-spectrum cephalosporin-resistant (CRE cases) and those with carbapenem-sensitive Enterobacterales (CSE controls). The study included a total of 226 participants (113 cases and 113 controls).

### Data collection

Data were obtained retrospectively from the report of data from the NHLS of the previous study on CRE: ‘Epidemiology, risk factors and molecular analysis of CRE in Mthatha, Eastern Cape, South Africa’. The collected data included demographics, organism(s) isolated, previous antimicrobial usage, medical records, medical history, among others. This data had already been collected in questionnaires (CRFs) from the main study with the help of a research assistant and medical practitioner. In the main study, participants had given consent with the allowance for the collected to be able to be used by studies in future or not. The CRF included the following sections: demographic-related factors, medical history-related factors and current hospitalisation-related factors.

### Data analysis

Comparisons were analysed between cases and controls. Variables of investigations in laboratory reports were captured and coded in Microsoft Excel, and then transferred to GraphPad Prism version 8 software for analysis. Categorical variables were presented using frequency tables and percentages. Data analysis included the use of ‘Chi-square test’ and ‘Fischer’s exact’ test to ascertain the association between the dependent variable (risk factors of CRE) and co-variables such as type of organism, antimicrobial resistance, medical records, CRE case and CRE control.

The evaluation was carried out at a 95% confidence interval, and the Baptista-Pike method was used to compute confidence intervals and odd ratios. *P*-value ≤ 0.05 was considered statistically significant. Assistance from statisticians was sought.

### Microbiological investigation

Enterobacterales isolates were locally identified by the system used in NHLS, Mthatha, identification (ID) and AST-Vitek2^®^ system (bioMérieux, Marcy l’Etoile, France). The detection of *bla*_OXA__-48_, *bla*_KPC_, *bla*_NDM_ and *bla*_VIM_ was carried out by the RESIST-4 O.K.N.V (lateral flow immunochromatographic assay). Susceptibility to antibiotics was determined by the method used in NHLS (Vitek2^®^ [bioMérieux]) and interpreted according to the Clinical and Laboratory Standards Institute (CLSI) M100 ED33:2020 guidelines.^17^ Isolates were considered resistant to carbapenems if they were categorised as intermediate, susceptible or resistant to at least one of the carbapenems tested, which included ertapenem, imipenem or meropenem.

The ID and AST-Vitek2^®^ system utilises both biochemical ID and antimicrobial susceptibility testing (AST) to identify and assess Enterobacterales isolates.

### Ethical considerations

Only retrospective records of bacterial isolates from the previous study were sought, and no personal or identifying information of the studied participants was included, as this was a retrospective study involving previously non-identified patients. The main study, ‘Epidemiology, risk factors and molecular analysis of Carbapenem-resistant Enterobacterales (CRE) in Mthatha, Eastern Cape, South Africa’ was approved by the Walter Sisulu University Health Research Ethics and Bio-safety Committee (080/2017) and the Eastern Cape Health Research Committee (EC_201710_010). The data had already been collected, extracted from the medical records and clinical research forms (CRFs), and stored in password-protected databases. This sub-study was also approved by Walter Sisulu University, with the Health Research Ethics and Bio-safety Committee approval number for the main study (HREC:080/2017) and the present study (HREC: 077/2023). To protect the identity of patients, laboratory information and information filled in the CRFs were coded in password-protected Microsoft Excel files.

## Results

### Participants eligibility inclusion and exclusion

**FIGURE 1:**
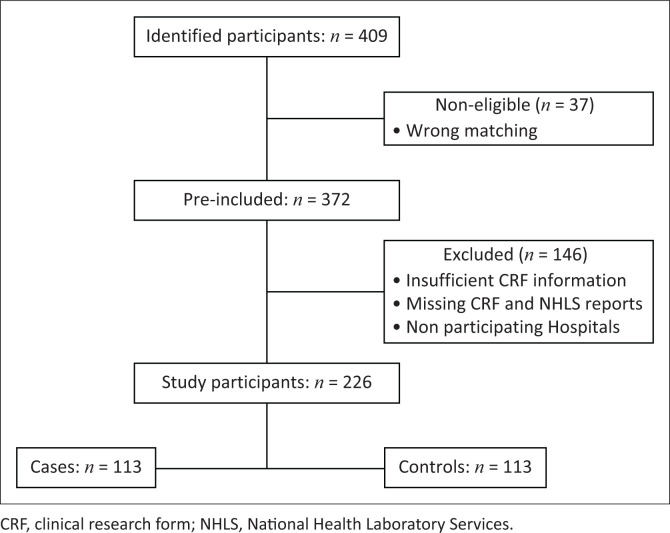
The eligibility, inclusion and exclusion of participants.

Out of the 409 study participants identified during the study period, 372 were initially considered for inclusion, while 37 participants were excluded from cases as they were incorrectly matched (non-CRE). Among the 372 initially included participants, 146 were further excluded because of reasons such as insufficient information on the CRF, missing CRFs, missing NHLS reports and participant admittance from other surrounding hospitals rather than NMAH and MRH. After the exclusions, 113 cases were successfully matched with 113 controls, resulting in a total of 226 study participants (Figure 1). The most common sample for cases was urine (31.0%), followed by wound swabs (20.4%). Conversely, the most common sample for controls was a blood culture (39.8%), followed by a wound swab (28.3%). Carbapenem-resistant Enterobacterales infection was confirmed clinically by the attending clinician.

Carbapenem-resistant Enterobacterales colonisation refers to the presence of CRE in a patient without causing an infection or clinical symptoms. In this state, the bacteria reside in or on the patient’s body but do not actively cause harm, although the individual remains at risk of developing a CRE infection if exposed to certain predisposing factors. In contrast, CRE infection involves clinical manifestations caused by bacteria entering the body, often through medical devices like urinary catheters, intravenous lines, ventilators or surgical wounds. This leads to active infections at specific sites within the body.

### Antimicrobial susceptibility analysis of isolated Enterobacterales and resistant genes

The antimicrobial susceptibility analysis of Enterobacterales isolates revealed high resistance rates to carbapenem antibiotics. For imipenem, 63.1% of isolates were resistant, meropenem showed 62.5% resistance and ertapenem had the lowest resistance rate at 50.4%. Among CRE cases, the most common resistance gene identified was *bla*_OXA-48_ (49.1%), followed by *bla*_NDM_ (12.3%), while 38.7% tested negative for resistance genes. No *bla*_KPC_ or *bla*_VIM_ genes were detected in the study (Table 1).

**TABLE 1a:** Antimicrobial susceptibility analysis of isolated Enterobacterales and resistant genes.

Antibiotic resistance	Imipenem (*n* = 111)		Meropenem (*n* = 112)		Ertapenem (*n* = 113)	
	*n*	%	*n*	%	*n*	%
S	25	22.5	15	13.4	30	26.5
I	16	14.4	27	24.1	26	23.0
R	70	63.1	70	62.5	57	50.4

S, Susceptible; I, Intermediate; R, Resistance.

**TABLE 1b:** Antimicrobial susceptibility analysis of isolated Enterobacterales and resistant genes.

Resistant gene	CRE cases† (*n* = 106)	%
*bla*_OXA-48_	52	49.1
*bla*_NDM_	13	12.3
Negative	41	38.7

Note: Additionally, no *bla*_KPC_ and *bla*_VIM_ genes were identified from participants in this study.

CRE, Carbapenem-resistant Enterobacterales.

† , Data were not available on resistant genes for seven case participants.

## Analyses of CRE cases against CSE controls

### Demographic-related factors associated with CRE isolates through case (participants with CRE isolate) – control (participants with CSE isolate) study

When comparing demographic-related factors, case participants were more likely than controls to be adults (odds ratio [OR]: 1.34, 95% confidence interval [CI]: 0.72–2.55) and case participants were likely to be males (54.9%). Additionally, compared to infants (≤ 1 year), cases were less likely to be paediatric participants (> 1 year to ≤ 19 years) (OR: 1.78, 95% CI: 0.79–4.10) or young adults (> 25 years to ≤ 65 years) (OR: 1.71, 95% CI: 0.63–4.91) and elderly (> 65 years) (OR: 0.72, 95% CI: 0.28–2.13). Case participants were most likely adults (36.6%). Regarding occupational status and travel history, case participants were more likely than control participants to be in retirement (OR: 1.11, 95% CI: 0.30–4.22) and to have travelled outside of the Eastern Cape province of South Africa (OR: 1.46, 95% CI: 0.84–2.56). Additionally, case participants were less likely than controls to be dependents (OR: 0.96, 95% CI: 0.36–2.55) or unemployed (OR: 0.75, 95% CI: 0.24–2.27). However, the analysis did not reveal statistically significant differences in demographic and lifestyle-related factors between CRE case participants and control participants (*p* > 0.05) (Table 2).

**TABLE 2:** Analysis of demographic-related factors associated with the development of Carbapenem-resistant Enterobacterales infections between case participants and control participants.

Variables	CRE case		CRE control		Cases			Controls			OR	95% CI	*p*
	*n*	%	*n*	%	*n*	*N*	%	*n*	*N*	%			
**Age (years)**	112	-	113	-	-	-	-	-	-	-	-	-	-
Infant	33	29.5	41	36.3	33	74	44.6	41	74	55.4	ref	-	-
Paediatrics	20	17.9	14	12.4	20	34	58.8	14	34	41.2	1.78	0.79–4.10	0.21
Young adult	11	9.8	8	7.1	11	19	57.9	8	19	42.1	1.71	0.63–4.91	0.32
Adults	41	36.6	38	33.6	41	79	51.9	39	79	48.1	1.34	0.72–2.55	0.42
Old age	7	6.3	12	10.6	7	19	36.8	12	19	63.2	0.72	0.28–2.13	0.61
**Sex**	113	-	113	-	-	-	-	-	-	-	-	-	-
Female	51	45.1	62	54.9	51	113	45.1	62	113	54.9	ref	-	-
Male	62	54.9	51	45.1	62	113	54.9	51	113	45.1	1.48	0.87–2.45	0.18
**Occupational status**	74	-	79	-	-	-	-	-	-	-	-	-	-
Working	9	12.2	9	11.4	9	18	50.0	9	18	50.0	ref	-	-
Unemployed	12	16.2	16	20.3	12	28	42.9	16	28	57.1	0.75	0.24–2.27	0.76
Retirement	10	13.5	9	11.4	10	19	52.6	9	19	47.4	1.11	0.30–4.22	> 0.99
Dependent	43	58.1	45	57.0	43	88	48.9	45	88	51.1	0.96	0.36–2.55	> 0.99
**Travel abroad/outside Eastern Cape**	89	-	102	-	-	-	-	-	-	-	-	-	-
Yes	44	49.4	41	40.2	44	85	51.8	41	85	48.25	1.46	0.84–2.56	0.24

OR, odds ratio; CI, confidence interval; CRE, Carbapenem-resistant Enterobacterales.

### Medical history-related factors associated with CRE isolates through case (participants with CRE isolate) – control (participants with CSE isolate) study

The analysis of medical-related factors between case participants and control participants revealed that a higher proportion of cases (58.8%) had underlying illnesses compared to controls (41.2%), (OR: 2.55, 95% CI: 1.41–4.60, *p* = 0.002). Chronic lung diseases were the most prevalent illnesses among cases, with 68.4% being positive for chronic pulmonary diseases, whereas only 31.6% of controls had chronic lung diseases. Additionally, more case participants (51.0%) had received medical care in the previous 12 months compared to controls who had also received medical care (49.0%), (OR: 1.29, 95% CI: 0.72–2.22). The majority of cases were admitted within 6 months from the onset of the study (56.9%) as compared to those who had been admitted beyond 6 months (43.1%) from the study onset.

More cases than controls had surgical or intensive care unit (ICU) admission instead of admission to the ward (OR: 2.39, 95% CI: 0.47–12.42).

Regarding carbapenem administration, more cases had previously received carbapenem antibiotics (57.1%) than controls (42.9%). Cases were more likely than controls to have a history of prior exposure to cephalosporin antibiotics (OR: 1.35, 95% CI: 0.30–6.98) followed by aminoglycosides (OR: 1.20, 95% CI: 0.25–6.39) compared to carbapenem antibiotics. More cases compared to controls were positive for HIV (OR: 1.20, 95% CI: 0.68–2.13) (Table 3).

**TABLE 3:** Analysis of medical history-related factors associated with the development of Carbapenem-resistant Enterobacterales infections between case participants and control participants.

Variables	CRE case		CRE control		Cases			Controls			OR	95% CI	*p*
	*n*	%	*n*	%	*n*	*N*	%	*n*	*N*	%			
**Underlying illness**	105	-	104	-	-	-	-	-	-	-	-	-	-
Yes	77	73.3	54	51.9	77	131	58.8	54	131	41.2	2.55	1.41–4.60	**0.002**
**Underlying illness (type)**	72	-	52	-	-	-	-	-	-	-	-	-	-
Cardiovascular disease	16	22.2	13	25.00	16	29	55.2	13	29	44.8	ref	-	-
Diabetes mellitus	6	8.3	10	19.23	6	16	37.5	10	16	62.5	0.48	0.15–1.57	0.35
Chronic lung disease	13	18.1	6	11.54	13	19	68.4	6	19	31.6	1.76	0.49–5.66	0.39
Renal disease	12	16.7	8	15.38	12	20	60.0	8	20	40.0	1.22	0.36–3.62	0.78
Neurologic disease	8	11.1	5	9.62	8	13	61.5	5	13	38.5	1.30	0.33–4.79	0.75
HIV	17	23.6	10	19.23	17	27	63.0	10	27	37.0	1.38	0.49–4.10	0.60
**Received medical care in the last 12 months**	93	-	101	-	-	-	-	-	-	-	-	-	-
Yes	51	54.8	49	48.5	51	100	51.0	49	100	49.0	1.29	0.72–2.22	0.39
If yes:	51	-	48	-	-	-	-	-	-	-	-	-	-
≤ 6 months	29	56.9	23	47.9	29	52	55.8	23	52	44.2	ref	-	-
˃ 6 months	22	43.1	25	52.1	22	47	46.8	25	47	53.2	0.70	0.33–1.57	0.42
**Medical care received (type)**	52	-	47	-	-	-	-	-	-	-	-	-	-
Admission to the ward	47	90.4	45	95.7	47	92	51.1	45	92	48.9	ref	-	-
SurgicalICU admission	5	9.6	2	4.3	5	7	71.4	2	7	28.6	2.39	0.47–12.42	0.44
**Previous antibiotics (last 6 months)**	78	-	89	-	-	-	-	-	-	-	-	-	-
Yes	34	43.6	34	38.2	34	68	50.0	34	68	50.0	1.25	0.69–2.28	0.53
If yes, Antibiotics (type)	34	-	34	-	-	-	-	-	-	-	-	-	-
Carbapenems	8	23.5	6	17.6	8	14	57.1	6	14	42.9	ref	-	-
Aminoglycoside	8	23.5	5	14.7	8	13	61.5	5	13	38.5	1.20	0.25–6.39	> 0.99
Penicillin	14	41.2	14	41.2	14	28	50.0	14	28	50.0	0.75	0.23–2.74	0.75
Cephalosporins	9	26.5	5	14.7	9	14	64.3	5	14	35.7	1.35	0.30–6.98	> 0.99
Other	8	23.5	10	29.4	8	18	44.4	10	18	55.6	0.60	0.15–2.36	0.72
**HIV status**	103	-	104	-	-	-	-	-	-	-	-	-	-
Positive	42	40.8	38	36.5	42	80	52.5	38	80	47.5	1.20	0.68–2.13	0.57

HIV, human immunodeficiency virus; OR, odds ratio; CI, Confidence interval; CRE, Carbapenem-resistant Enterobacterales; ICU, intensive care unit.

### Current hospitalisation-related factors associated with CRE isolates through case (participants with CRE isolate) – control (participants with CSE isolate) study

The analysis of current hospitalisation-related factors between case participants and control participants revealed that a higher proportion of cases had been admitted at MRH (OR: 1.50, 95% CI: 0.75–2.98) and more cases than controls were admitted in surgical wards (OR: 1.75, 95% CI: 0.91–3.26). Among the CRE species isolated, *Klebsiella pneumoniae* showed a significant prevalence (OR: 14.06, 95% CI: 5.22–34.32, *p* < 0.0001), followed by *Enterobacter cloacae* (OR: 12.92, 95% CI: 3.93–36.33, *p* < 0.0001). More case participants (53.6%) had hospital-acquired infections than controls (46.4%), and hospital-acquired infections were more prevalent than community-acquired infections (OR: 1.21, 95% CI: 0.61–2.42). Bloodstream infections were the most prevalent hospital-acquired infections in case participants compared to control participants (OR: 2.37, 95% CI: 0.96–6.31), and cases were more likely than controls to be colonised (OR: 1.56, 95% CI: 0.70–3.36).

Analysis of current CRE risk factors showed that cases were more likely than controls to have urine catheterisation (OR: 5.40, 95% CI: 1.45–18.33, *p* = 0.01), infection in the preceding 3 months (OR: 3.96, 95% CI: 1.15–14.23), insertion of intravascular devices (OR: 2.48, 95% CI: 1.06–6.03, *p* = 0.05) and to have been given antibiotics between admission and study inclusion (OR: 1.51, 95% CI: 0.31–8.65). Among the administered antibiotics, cases were more likely to have been administered fluoroquinolones (OR: 2.17, 95% CI: 0.87–5.75), polymyxins (OR: 1.71, 95% CI: 0.64–4.45) and glycopeptides (OR: 1.16, 95% CI: 0.48–2.86) than carbapenem antibiotics.

Regarding the final outcomes following hospitalisation, cases (32.0%) were significantly more likely than controls (15.1%) to have demised during the study period and to have experienced a prolonged hospital stay exceeding 1 month (OR: 1.87, 95% CI: 1.01–3.39, *p* = 0.048) (Table 4).

**TABLE 4:** Analysis of current hospitalisation-related factors associated with the development of Carbapenem-resistant Enterobacterales infections between case participants and control participants.

Variables	CRE case		CRE control		Cases			Controls			OR	95% CI	*p*
	*n*	%	*n*	%	*n*	*N*	%	*n*	*N*	%			
**Hospital**	113	-	113	-	-	-	-	-	-	-	-	-	-
NMAH	88	77.9	95	84.1	88	183	48.1	95	183	51.9	ref	-	-
MRH	25	22.1	18	15.9	25	43	58.1	18	43	41.9	1.50	0.75–2.98	0.31
**Ward level**	113	-	111	-	-	-	-	-	-	-	-	-	-
Medical ward (s)	36	31.9	44	39.6	36	80	45.0	44	80	55.0	ref	-	-
Surgical ward(s)	40	35.4	28	25.2	40	68	58.8	28	68	41.2	1.75	0.91–3.26	0.10
High risk (s)	37	32.7	39	35.1	37	76	48.7	39	76	51.3	1.16	0.62–2.16	0.75
**CRE species isolated**	113	-	113	-	-	-	-	-	-	-	-	-	-
*Escherichia coli* (ESCCO)	5	4.4	42	37.2	5	47	10.6	42	47	89.4	ref	-	-
*Klebsiella pneumoniae* (KLEPP)	77	68.1	46	40.7	77	123	62.6	46	123	37.4	14.06	5.22–34.32	**˂ 0.0001**
*Enterobacter cloacae* (ENTCL)	20	17.7	13	11.5	20	33	60.6	13	33	39.4	12.92	3.93–36.33	**˂ 0.0001**
*Serratia marcescens* (SERMA)	3	2.7	13	11.5	3	16	18.75	13	16	81.25	1.94	0.46–9.22	0.41
**HAI**	109	-	98	-	-	-	-	-	-	-	-	-	-
Yes	89	81.7	77	78.6	89	166	53.6	77	166	46.4	1.21	0.61–2.42	0.60
If yes, HAI (type)	90	-	65	-	-	-	-	-	-	-	-	-	-
RTI	16	17.8	17	26.2	16	33	48.5	17	33	54.5	ref	-	-
CAUTI	26	28.9	17	26.2	26	43	60.5	17	43	39.5	1.63	0.68–3.98	0.356
SSI	19	21.1	18	27.7	19	37	51.4	18	37	48.6	1.12	0.45–2.84	> 0.99
BSI	29	32.2	13	20.0	29	42	69.0	13	42	31.0	2.37	0.96–6.31	0.10
**Current CRE risk factors:**	99	-	83	-	-	-	-	-	-	-	-	-	-
Mechanical ventilation	10	10.1	18	21.7	10	28	35.7	18	28	64.3	ref	-	-
Urine catheterisation	15	15.2	5	6.0	15	20	75.0	5	20	25.0	5.40	1.45–18.33	**0.01**
Intravascular devices	55	55.6	40	48.2	55	95	57.9	40	95	42.1	2.48	1.06–6.03	**0.05**
surgery during preceding 6 months	8	8.1	15	18.1	8	23	34.8	15	23	65.2	0.96	0.33–3.27	> 0.99
Infection in the preceding 3 months	11	11.1	5	6.0	11	16	68.8	5	16	31.3	3.96	1.15–14.23	0.06
**Antibiotic prescription**	109	-	109	-	-	-	-	-	-	-	-	-	-
Yes	107	98.2	106	97.2	107	213	50.2	106	213	49.8	1.51	0.31–8.65	> 0.99
**Current antibiotic prescription**	107	-	106	-	-	-	-	-	-	-	-	-	-
Carbapenems	35	32.7	38	35.8	35	73	47.9	38	73	52.1	ref	-	-
Aminoglycosides	45	42.1	51	48.1	45	96	46.9	51	96	53.1	0.96	0.51–1.76	> 0.99
Penicillin	55	51.4	64	60.4	55	119	46.2	64	119	53.8	0.93	0.52–1.67	0.88
Cephalosporins	28	26.2	33	31.1	28	61	45.9	33	61	54.1	0.92	0.48–1.79	0.86
Glycopeptides	15	14.0	14	13.2	15	29	51.7	14	29	48.3	1.16	0.48–2.86	0.83
Polymyxin	11	10.3	7	6.6	11	18	61.1	7	18	38.9	1.71	0.64–4.45	0.43
Macrolides	5	4.7	9	8.5	5	14	35.7	9	14	64.3	0.60	0.21–20.4	0.56
Fluoroquinolones	16	15.0	8	7.5	16	24	66.7	8	24	16	-	-	15.0
**Final patient outcome**	97	-	86	-	-	-	-	-	-	-	-	-	-
Discharged home	58	59.8	56	65.1	58	114	50.9	56	114	49.1	ref	-	-
Transferred to step-down hospital	8	8.2	17	19.8	8	25	32.0	17	25	68.0	0.45	0.19–1.13	0.12
Demised	31	32.0	13	15.1	31	44	70.5	13	44	29.5	2.30	1.10–4.71	**0.03**
**Length of hospital stay**	92	-	85	-	-	-	-	-	-	-	-	-	-
< 1 month	48	52.2	57	67.1	48	105	45.7	57	105	54.3	ref	-	-
> 1 month	44	47.8	28	32.9	44	72	61.1	28	72	39.9	1.87	1.01–3.39	**0.05**

OR, odds ratio; CI, confidence interval; CRE, Carbapenem-resistant Enterobacterales; NMAH, Nelson Mandela Academic Hospital; MRH, Mthatha Regional Hospital; HAIs, healthcare-associated infections; CAUTI, catheter-associated urinary tract infection; RTI, respiratory tract infection; SSI, surgical site infection; BSI, bloodstream Infection.

## Discussion

Carbapenem-resistant Enterobacterales and its related factors pose a serious threat to public health because of their association with significant morbidity and mortality globally.^2,3^ Worldwide, CRE are significant pathogens within healthcare settings and are accountable for dire infections among the public.^18^ In Mthatha, South Africa, there is limited data available regarding the risk factors associated with the development of CRE infections. Therefore, this study investigated risk factors associated with the development of CRE infections among participants who received medical care from NMAH and MRH in Mthatha, Eastern Cape. This study revealed that the majority of patients were adults (36.6%), followed by infants (29.5%). This distribution may be attributed to the susceptibility of individuals in these age groups to pathogen invasion because of factors such as diminishing immunity in elderly adults and immature immunity in infants. Regarding sex, this study revealed that most case patients were males (54.9%). This finding is consistent with previous studies examining risk factors for CRE infections as seen in a case-control analysis by Nicolas-Chanoine et al.^19^ in French hospitals which also reported that 68.0% of case patients were males. Similarly, a study conducted in South Africa on CRE in patients with bacteraemia at tertiary hospitals by Perovic et al.^8^ reported that male patients accounted for 54% of cases compared to female cases.

Regarding medical history-related factors, this study revealed that a significant proportion of cases compared to controls had underlying illnesses (*n* = 77/105 [73.3%] vs. *n* = 54/104 [51.9%]; *p* = 0.002; odds ratio [OR] = 2.55 [1.41–4.60]). This suggests that patients with underlying illnesses such as chronic lung diseases, HIV positivity, neurologic diseases and renal diseases are at an increased risk of developing CRE infections. These findings align with previous studies indicating that underlying illnesses or conditions are risk factors associated with acquiring CRE infections.^20^ Additionally, an analysis of risk factors for CRE infections conducted by Gao et al.^14^ highlighted underlying diseases as a risk factor for CRE infections. Patients with underlying illnesses are at a higher risk of CRE infections because of weakened immunity, which makes them susceptible to pathogenic microorganisms. A systematic review conducted by Palacios-Baena et al.^21^ highlighted a significant association between CRE infections and previous antibiotic exposure. Including association with previous carbapenem use, this was also previously noted by Asai et al.^22^ However, this study did not reveal a significant association of CRE infection with previous antibiotic exposure. This discrepancy may stem from the notion that antibiotics were overused in all patients in NMAH and MRH including CRE control patients.

Nevertheless, this study found that more cases than controls had previous carbapenem use (*n* = 8/14 [57.1%] vs. *n* = 6/14 [42.9%]), consistent with the findings of Palacios-Baena et al.^21^ and in line with reports from studies by Van Loon et al.^20^ and Marimuthu et al.^23^ Patients who have been previously exposed to antibiotics and specifically carbapenems are at a heightened risk of developing CRE infections, as microorganisms may develop resistance to carbapenem antibiotics because of the misuse or overuse of these antibiotics.

Analysis of current hospitalisation-related factors in this study revealed that cases were more likely than controls to have been admitted to surgical wards (*n* = 40/68 [58.8%] vs. *n* = 28/68 [41.2%]). Consistent with these findings, a previous study conducted among patients in an acute tertiary hospital in Singapore also reported admission to surgical ICU as a risk factor associated with CRE.^24^ Similarly, a systematic review of risk factors for CRE acquisition and clinical outcomes of CRE infections in Africa identified admission to the surgical ICU as one of the frequent factors associated with CRE infection.^25^ Additionally, an analysis of patients admitted to surgical wards revealed that surgical procedures were found to be risk factors for CRE.^26^

Microbiological results in this study revealed that a significant proportion of Enterobacterales species detected in cases was *Klebsiella pneumoniae* (*n* = 77 of 113, 68.1%), followed by *Enterobacter cloacae* (*n* = 20 of 113, 17.7%). When the presence of these species was compared between cases and controls, *K. pneumoniae* accounted for 62.6% (*p* < 0.0001) and *E. cloacae* accounted for 60.6% (*p* < 0.0001) among cases. These findings are consistent with a study conducted in a tertiary hospital in Johannesburg, South Africa, which reported that the predominant members of the Enterobacterales family identified as CRE were *K. pneumoniae*, followed by *E. cloacae*.^27^ Similarly, a study conducted in three tertiary care hospitals in China reported that the most common CRE pathogens identified were *K. pneumoniae* (67.7%) and *E. cloacae* (19.4%).^28^ Additionally, a prospective study on the treatment of CRE infection and risk factors associated with outcomes, conducted by de Maio Carrilho et al.,^29^ reported *K. pneumoniae* and Enterobacter spp. as the most common CRE pathogens detected.

This study investigated current hospitalisation-related factors and identified urinary catheterisation (*n* = 15/20 [75.0%] vs. *n* = 5/20 [25.0%]; *p* = 0.01; OR = 5.40 [1.45–18.33]) and intravascular devices (*n* = 55/95 [57.9%] vs. *n* = 40/95 [42.1%]; *p* = 0.05; OR = 2.48 [1.06–6.03]) as significant factors associated with CRE infections among cases as compared to controls.

Similarly, a study by Tian et al.^30^ also highlighted urinary system diseases as risk factors associated with CRE infections. Also, the findings from Gao et al.^14^ revealed a notable association between patients who underwent invasive procedures and an increased risk of developing CRE infection. This association was also observed in the results of a prior investigation conducted by Lodise et al.^31^ in 2017, highlighting that around 10% of invasive procedures pose a risk for CRE infection. Similarly, Wang’s study,^32^ focussing on patients with CRE infection, identified tracheal intubation as a factor contributing to the risk of CRE infection. This is because patients with urinary obstruction or incontinence often require medical devices, such as urinary catheters which can increase the likelihood of bacterial invasions.^33^ Moreover, patients with UTIs are treated with prolonged or multiple courses of antibiotics, which can result in long-term alterations in the normal microbiota of the gastrointestinal tract and the emergence of multi-drug resistant (MDR) microorganisms.^34^ Additionally, invasive procedures elevate the susceptibility to CRE infections because of the compromised immune state of patients. Improper administration of these procedures facilitates bacterial entry through wounds, leading to potential complications and the onset of CRE infections.^14^ Previous research has indicated that the fatality rate among patients with CRE infections typically ranges from 30% to 75%.^35,36,37^ Patients with CRE infections were significantly more likely to die during hospitalisation compared to patients with CSE infections (*n* = 31/97 [32.0%] vs. *n* = 13/86 [15.1%]; *p* = 0.03; OR = 2.30 [1.10–4.71]) consistent with the findings observed in earlier studies.

Several factors contribute to the high mortality rate, including prolonged hospital stays, poor overall health status, the site of infection, the presence of comorbid illnesses, and notably, the limited availability of effective antimicrobial treatment options for addressing these infections.^38^ This study found that a significant proportion of cases experienced prolonged hospital stays of more than 1 month (*n* = 44/72 [61.1%] vs. *n* = 28/72 [39.9%]; *p* = 0.05; OR = 1.87 [1.01–3.39]). Similarly, findings from the study by Asai et al.^22^ reported that participants from the CRE case group tended to have longer hospital stays (121 days vs. 63 days, *p* = 0.052) than participants from the control group. A study conducted by Adams et al.^39^ also reported that patients hospitalised with CRE infection had longer hospital stays (attributable difference, 28.8 days; *p* < 0.001). Extended hospital stays elevate the probability of encountering opportunistic pathogens within the hospital environment, leading to a higher incidence of CRE colonisation and subsequent infection.

### Treatment and management of CRE infections

Treatment options for CRE include various antibiotics such as carbapenems (if the isolate is sensitive), cefiderocol, polymyxins, aminoglycosides, tigecycline, fluoroquinolones and BLBLI such as ceftazidime-avibactam. In addition to antibiotic therapy, effective management strategies emphasise antimicrobial stewardship, routine screening and surveillance, strict hand hygiene and proper sterilisation of medical equipment. Patient isolation is also essential to prevent the spread of CRE infections within healthcare settings.^40^

### Limitations, strengths and generalisability

This study has some limitations. Firstly, the research was conducted in only two hospitals in Mthatha, which may limit the generalisability of the findings to other regions of the Eastern Cape or South Africa as a whole. Secondly, the time constraints and challenges posed by the coronavirus disease 2019 (COVID-19) pandemic, particularly during lockdown periods, further complicated the completion of CRFs by healthcare workers during the main study and this has indirectly affected the sample size for this present study. Additionally, another limitation was the retrospective nature of the study, which relied on previously recorded data and may have introduced reporting biases.

Despite these limitations, the study has significant strengths. The use of matched case-control methodology ensured a thorough analysis of risk factors by controlling for confounding variables such as age, hospital and ward. The study also utilised molecular analysis of CRE isolates, providing detailed insights into resistance patterns and mechanisms, which are critical for informing clinical practice and policy development.

In terms of generalisability, while the findings are most directly applicable to the study hospitals, they offer valuable insights into CRE infections in similar settings with comparable healthcare challenges. The results can guide future studies and interventions aimed at reducing CRE infections in resource-limited healthcare environments.

## Conclusion

The study findings indicate that *Klebsiella pneumoniae* and *Enterobacter cloacae* were the most prevalent members of the Enterobacterales family identified as CRE, among patients admitted in NMAH and MRH in Mthatha, Eastern Cape, South Africa. The study suggests that underlying illnesses, urine catheters, invasive procedures and prolonged hospital stays significantly increase the likelihood of patients being susceptible to pathogenic microorganisms, leading to the development of CRE infections. Other contributing factors include admission to surgical ICUs, previous carbapenem use, receipt of medical care in the 12 months preceding the current admission and travel outside the Eastern Cape or abroad. Common carbapenemase genes identified in both hospitals were *bla*_OXA-48_ followed by *bla*_NDM_. Mortality rates were higher among CRE case patients as compared to CRE controls, and CRE cases were nearly twice as likely as controls to experience mortality or a prolonged hospital stay of more than 1 month (44 cases vs. 28 controls).
